# Depression is associated with lower adherence to cardioprotective medications in adults with type 1 diabetes

**DOI:** 10.1007/s00592-025-02610-3

**Published:** 2025-11-18

**Authors:** Raija Lithovius, Stefan Mutter, Erika B. Parente, Valma Harjutsalo, Per-Henrik Groop, Lena M. Thorn, Niina Sandholm

**Affiliations:** 1 Folkhälsan Research Center, Haartmaninkatu 8, 00290 Helsinki, Finland; 2 Department of Nephrology, University of Helsinki and Helsinki University Hospital, Helsinki, Finland; 3 Research Program for Clinical and Molecular Metabolism, Faculty of Medicine, University of Helsinki, Helsinki, Finland; 4 Boehringer Ingelheim International GmbH, Ingelheim, Germany; 5 Chronic Disease Prevention Unit, National Institute for Health and Welfare, Helsinki, Finland; 6 Department of Diabetes, Central Clinical School, Monash University, Melbourne, VIC Australia; 7 Baker Heart and Diabetes Institute, Melbourne, VIC Australia; 8 Department of General Practice and Primary Health Care, University of Helsinki and Helsinki University Hospital, Helsinki, Finland

**Keywords:** Type 1 diabetes, Medication adherence, Cardioprotective agents, Depression, Antidepressive agents

## Abstract

**Aims:**

To investigate the association between depression and refill adherence to cardioprotective medications in a representative cohort of type 1 diabetes adults.

**Methods:**

This Finnish Diabetic Nephropathy (FinnDiane) sub-study included 1,588 adults with type 1 diabetes who had purchased antihypertensive or lipid-lowering drugs within ± 0.5 years from study baseline. The proportion of days covered (PDC) method was used to calculate overall refill adherence over a 10-year follow-up. Adherence was classified into good (> 80%), intermediate (≥ 50 and < 80%), and poor (< 50%). Participants were considered to have depression, if they had a diagnosis of depression or had purchased antidepressive agents at any time point from 1995 until the end of follow-up, identified from national registries. Multinomial logistic regression analysis, adjusted for age, sex, duration of diabetes, education level, HbA_1c_, BMI, diabetic kidney disease, smoking, and alcohol consumption was performed.

**Results:**

Of the cohort 37% had depression during the study period. Those individuals with depression were more often women (*P* = 0.0001), and had lower adherence to cardioprotective medication (*P* = 0.02) than those without depression. Individuals with depression had 72% higher odds ([95% CI 1.09, 2.70], *P* = 0.02) of having poor versus good adherence, compare to those without depression. The results persisted after excluding antidepressants potentially used for neuropathic pain (OR 1.61 [95% CI 1.02, 2.55], *P* = 0.04).

**Conclusions:**

Healthcare professionals should assess presence of depression and monitor medication adherence at regular consultations, and whenever needed, should implement strategies to manage the depression in order to enhance adherence, which might ultimately lead to improved cardiovascular outcomes in type 1 diabetes.

**Supplementary Information:**

The online version contains supplementary material available at 10.1007/s00592-025-02610-3.

## Introduction

Cardiovascular disease (CVD) is a leading cause of morbidity and mortality in individuals with type 1 diabetes. As these individuals are vulnerable to developing CVD early in life, their life expectancy remains 10 to 12 years shorter than of the general population [1, 2]. Pharmacological therapies to control blood pressure (BP) and lipids play a key role in the prevention and slowing down of the progression of CVD and thereby improving outcomes [3]. For optimal pharmacological interventions, it is important to recognize those factors which may jeopardize adherence to cardioprotective medication. Depression is a common comorbidity in type 1 diabetes with up to 30% of individuals with type 1 diabetes displaying depressive symptoms [4]. Depression in individuals with type 1 diabetes is associated with poor glycaemic control [5], diabetic kidney disease (DKD) [6], and CVD [7]. Data are, however, limited to whether depression influences the adherence to cardioprotective treatment in individuals with type 1 diabetes. Therefore, in this study we aimed to evaluate the association between depression and overall refill adherence to cardioprotective medication in adults with type 1 diabetes.

## Methods

### The study cohort

The current study is part of the ongoing, nationwide multicenter Finnish Diabetic Nephropathy (FinnDiane) Study, established in 1997 to assess complications of diabetes in individuals with type 1 diabetes. The study protocol was approved by the Ethics Committee of the Helsinki and Uusimaa Hospital District, and the study was carried out in accordance with the Declaration of Helsinki. Written informed consent was obtained from each participant. A detailed FinnDiane protocol has been published previously [8]. Based on the national guidelines [9], alcohol consumption was classified into low (men < 14.0 doses/week, women < 7.0 doses/week, 1 dose contains 12 g of alcohol), moderate (men ≥ 14.0 & <23.0 doses/week, women ≥ 7.0 & <12.0 doses/week) and high (men ≥ 23.0 doses/week, women ≥ 12.0 doses/week) consumption. DKD was defined based on albuminuria in two out of three urine collections as moderate (albumin excretion rate [AER] ≥ 20 and < 200 µg/min or ≥ 30 and < 300 mg/24 h or albumin-creatinine ratio [ACR] ≥ 3.0 and < 30 mg/mmol] or severe albuminuria (AER ≥ 200 µg/min or ≥ 300 mg/24 h or ACR ≥ 30 mg/mmol), or estimated glomerular filtration rate (eGFR) < 60 ml/min/1.73 m^2^. Individuals on kidney replacement therapy at baseline, defined as dialysis or kidney transplantation, were excluded from the study.

### Refill adherence

Based on the information obtained from the Finnish Drug Prescription Register (DPR), maintained by the Social Insurance Institution of Finland, we included 1,588 adults with type 1 diabetes, who had filled prescriptions (i.e., purchased) for antihypertensive and/or lipid-lowering medications (Electronic Supplementary Material [ESM] Table S1) for the first time within half a year before or after the baseline study visit [i.e., index date] and had at least two purchases during at least one year of follow-up. The proportion of the days covered (PDC) method was used to calculate the overall refill adherence, defined as the percentage of days that an individual had access to all medication subgroups in their treatment regimen [10, 11]. Refill adherence was calculated from baseline (i.e., from index date) over the 10-year follow-up period. In line with other studies and our own previous research on adherence to cardioprotective drugs and CVD, we classified adherence into good (≥ 80%), intermediate (≥ 50 and < 80%), or poor (< 50%) adherence [11, 12].

### Definition of depression

The occurrence of depression, was defined either by a diagnosis of depression based on any depression-related hospital visits (International Cla*s*sification of Diseases [ICD]-9 codes 2961 and 2968; ICD-10 codes F32, F33, and F34.1), registered in the Finnish Care Register for Health Care, maintained by the Finnish Institute for Health and Welfare, or by a purchase of antidepressive agents obtained from the DPR (ATC-codes: N06A, N06CA), at any time point during the median study period of 15.9 (Interquartile range [IQR] 13.8–18.9) years. History of depression was defined as having at least one depressive episode between 1995 and the study baseline visit without a relapse after baseline. By contrast, new onset depression was defined as having the first depressive episode during the 10-year follow-up period after the baseline. Persistent depression was defined as having depression episodes both before and after the baseline. The primary outcome measure of this study was any occurrence of depression which combines a history of depression, persistent depression and new onset cases. We also conducted sensitivity analysis by relying only on ICD codes for depression diagnosis, and excluding the use of antidepressants, which can be used for other indications such as neuropathic pain, anxiety, and insomnia (ESM Table S2).

### Statistical analysis

Data presented are mean ± standard deviation, median (IQR), or percentages. Differences between the groups were tested for normally distributed variables with *t*-test and for non-normally distributed variables with Wilcoxon test. Differences in categorical variables were assessed with Pearson’s chi-squared test or two-tailed Fisher’s exact test. Furthermore, in order to investigate the association between depression and adherence categories, multinomial logistic regression analysis was carried out adjusted in the full model for age, sex, duration of diabetes, education level, glycated haemoglobin (HbA_1c_), body mass index (BMI), DKD, smoking, and alcohol consumption. Statistical analyses were performed with the R statistical software [13] version 4.4.2.

## Results

Overall, 37% of the study cohort had depression during the median study period of 15.9 (IQR13.8–18.9) years. Of them, 3% had a depression diagnosis only, 67% had purchases of antidepressants only, and 30% had both a diagnosis and purchases of antidepressants. Table 1 presents the clinical characteristics of individuals with history, new onset, persistent or any depression. Those with any depression were more often women (50.3% vs. 40.2%, *P* = 0.0001), and had lower median adherence (73.8% vs. 75.0%, *P* = 0.02) and slightly higher waist-to-height ratio (*P* = 0.03) than those without depression. Moreover, 36.3% of those with any depression had good, 53.9% intermediate, and 9.8% poor adherence to cardioprotective medications during the 10-year assessment period following the study baseline visit. Interestingly,* p*articipants with new onset depression had the lowest median adherence (72.0%), which was significantly lower than in those without depression (*P* = 0.01). However, adherence among those with history of depression (*P* = 0.5), or persistent depression (*P* = 0.4) was similar to that of individuals without depression We did not find any baseline differences with regards to glycaemic control, smoking, or DKD between adults with and without any depression. Notably, those with new onset depression had higher alcohol consumption compared to those without depression (*P* = 0.04).

**Table 1:** Baseline characteristics of individuals with type 1 diabetes according to their depression status (*N* = 1,588)

Depression status	Never	History of depression	New onset depression	Persistent depression	Any depression	*P*-value			
Variable						History vs. never	New onset vs. never	Persistent vs. never	Any vs. never
N (%)	998 (62.8)	143 (9.0)	333 (21.0)	114 (7.2%)	590 (37.2)				
Adherence %, q1–q3	75.0 (64.5–85.2)	75.9 (61.1–85.2)	72.0 (60.7–84.2)	74.9 (61.5–83.0)	73.8 (61.4–85.1)	0.5	0.01	0.4	0.02
Adherence categories						0.2	0.1	0.6	0.2
Good, n (%)	380 (38.1)	59 (41.3)	116 (34.8)	39 (34.2)	214 (36.3)				
*Intermediate*, n (%)	545 (54.6)	69 (48.2)	181 (54.4)	68 (59.7)	318 (53.9)				
Poor, n (%)	73 (7.3)	15 (10.5)	36 (10.8)	7 (6.1)	58 (9.8)				
Age (years)	42.4 ± 11.1	45.*1* ± 10.6	41.7 ± 10.0	45.0 ± 10.8	43.1 ± 10.5	0.007	0.2	0.02	0.2
Female, n (%)	401 (40.2)	80 (55.9)	154 (46.2)	63 (55.3)	297 (50.3)	0.0005	0.06	*0.003*	0.0001
Duration of diabetes, years	26.7 ± 10.6	29.4 ± 10.6	26.3 ± 10.3	29.5 ± 11.2	27.7 ± 10.6	0.005	0.5	0.01	0.09
Age at onset of diabetes, years	13.5 (8.6–22.2)	13.5 (9.2–20.9)	13.1 (8.8–20.8)	13.2 (8.3–23.1)	13.2 (8.9–21.4)	0.9	0.6	0.7	0.7
University level of education, n (%)	309 (35.8)	53 (42.*4*)	91 (30.5)	33 (33.3)	177 (33.9)	0.2	0.1	0.7	0.5
Albuminuria status						0.09	0.4	0.01	0.1
Normal AER n (%)	403 (40.4)	70 (48.9)	134 (40.2)	60 (52.6)	264 (44.7)	0.06	1.0	0.02	0.1
Moderate albuminuria, n (%)	256 (25.6)	36 (25.2)	75 (22.5)	17 (14.9)	128 (21.7)	1.0	0.3	0.02	0.09
Severe albuminuria, n (%)	339 (34.0)	37 (25.9)	124 (37.2)	37 (32.5)	198 (33.6)	0.07	0.3	0.8	0.9
eGFR, ml min^− 1^(1.73 m)^−2^	91.5 (69.8–107.0)	95.0 (71.1–107.3)	92.3 (62.4–107.3)	91.2 (72.2–104.9)	92.4 (66.8–107.3)	0.4	0.4	1.0	0.7
Diabetic kidney disease, n (%)	598 (60.0)	74 (52.1)	205 (61.9)	56 (49.2)	335 (57.1)	0.09	0.6	0.03	0.3
Systolic BP, mmHg	142 ± 18	140 ± 19	141 ± 19	140 ± 16	140 ± 18	0.2	0.4	0.2	0.1
Diastolic BP, mmHg	82 ± 10	80 ± 10	82 ± 10	80 ± 10	81 ± 10	0.05	0.9	0.07	0.2
BMI, kg/m^2^	25.9 ± 3.6	25.8 ± 3.9	26.3 ± 4.0	26.6 ± 4.4	26.2 ± 4.1	0.7	0.1	0.1	0.1
Waist-to-height ratio	0.52 ± 0.06	0.53 ± 0.07	0.53 ± 0.06	0.53 ± 0.08	0.53 ± 0.07	0.3	0.1	0.06	0.03
Total cholesterol, mmol/l	5.1 ± 1.0	5.0 ± 0.9	5.2 ± 1.1	5.2 ± 0.9	5.2 ± 1.0	0.4	0.07	0.5	0.2
HDL cholesterol, mmol/l	1.3 ± 0.4	1.5 ± 0.4	1.3 ± 0.4	1.4 ± 0.4	1.4 ± 0.4	< 0.0001	0.5	0.1	0.005
LDL cholesterol, mmol/l	3.2 ± 0.9	3.0 ± 0.9	3.3 ± 0.9	3.2 ± 0.8	3.2 ± 0.9	0.02	0.3	0.6	0.8
Triglycerides mmol/l	1.1 (0.8–1.6)	1.1 (0.8–1.5)	1.1 (0.9–1.7)	1.1 (0.8–1.7)	1.1 (0.8–1.7)	0.3	0.5	0.8	0.8
HbA_1c_, %	8.6 ± 1.4	8.5 ± 1.4	8.7 ± 1.4	8.6 ± 1.5	8.6 ± 1.4	0.5	0.1	0.7	0.3
HbA_1c_, mmol/mol	70 ± 15	69 ± 16	72 ± 16	71 ± 17	71 ± 16	0.5	0.1	*0.7*	0.3
Current smoker, n (%)	217 (23.1)	36 (25.7)	80 (25.3)	22 (24.4)	138 (24.5)	0.6	0.5	0.6	0.6
Alcohol consumption						0.3	0.04	0.05	0.07
Low, n (%)	790 (90.4)	104 (89.6)	243 (85.0)	87 (86.1)	434 (86.3)				
Moderate, n (%)	59 (6.7)	11 (9.5)	31 (10.8)	6 (5.9)	48 (9.5)				
High, n (%)	25 (2.9)	1 (0.9)	12 (4.2)	8 (7.9)	21 (4.2)				

Differences between the groups were tested for normally distributed variables with t-test and for non-normally distributed variables with Wilcox signed rank test. Differences in distributions were tested with Pearson’s chi-squared test or two-tailed Fisher exact test. Data are mean ± standard deviation, median (IQR), or %. *P*-values indicate comparisons with individuals who neither had diagnosed depression nor purchases of antidepressants during the study period

Results from the multinomial regression analysis are presented in Table 2. Individuals with any depression had 51% higher odds (95% CI 1.02, 2.25, *P* = 0.04) of having poor adherence versus good adherence, when adjusted for age, duration of diabetes, sex, BMI, HBA_1c,_ and presence of kidney complications at baseline, compared to those without depression. The results remained significant after additional adjustments for smoking, alcohol consumption, and education level (OR 1.72 [95% CI 1.09, 2.70], *P* = 0.02). Similarly, in the sensitivity analysis, when we excluded antidepressants, which are potentially used for the treatment of neuropathic pain, the results did not change even after full adjustment (OR 1.61 [95% CI 1.02, 2.55], *P* = 0.04). However, there was no significant association between depression status and having intermediate adherence compared to good adherence (OR 1.06 [95% CI 0.83, 1.36],  *P*= 0.6).

**Table 2:** Associations between any depression and adherence classes for cardioprotective medications in individuals with type 1 diabetes (multinomial regression analysis with adherence as outcome variable and any depression as explanatory variable)

Adherence class	Any depression no/yes *n (%)*	Model 1^b^		Model 2^c^		Model 3^d^		
		OR (95% CI), P	z	OR (95% CI), P	z	OR (95% CI), P	z	
Good	380 (64.0) / 214 (36.0)	Ref.		Ref.		Ref.		
Intermediate	545 (63.2) / 318 (36.8)	1.04 (0.83, 1.29), 0.7	0.3198	1.06 (0.85, 1.32), 0.6	0.4764	1.06 (0.83, 1.36), 0.6	0.4869	
Poor	73 (55.7) / 58 (44.3)	1.41 (0.96, 2.07), 0.08	1.7596	1.51 (1.02, 2.25), 0.04	2.0396	1.72 (1.09, 2.70), 0.02	2.3475	
Good^*a*^	407 (68.5) / 187 (31.5)	Ref.		Ref.		Ref.		
Intermediate^*a*^	591 (68.5) / 272 (31.5)	1.00 (0.80, 1.25), 1.0	0.0146	1.03 (0.82, 1.29), 0.8	0.2374	1.05 (0.82, 1.36), 0.7	0.3995	
Poor^*a*^	81 (61.8) / 50 (38.2)	1.34 (0.91, 1.99), 0.1	1.4733	1.43 (0.95, 2.14), 0.08	1.7236	1.61 (1.02, 2.55), 0.04	2.0366	

^*a*^Antidepressants potentially prescribed for neuropathic pain i.e., Amitriptyline (Anatomical Therapeutic Chemical [ATC] N06AA09), Nortriptyline (N06AA10), Venlafaxine (N06AX16), Duloxetine (N06AX21) and Amitriptyline combination (N06CA01) excluded

^*b*^ Unadjusted

^*c*^Adjusted for age, sex, duration of diabetes, BMI, HbA_1c_, kidney complications (moderate or severe albuminuria or eGFR < 60 ml/min/1.73 m^2^)

^*d*^Model 2 + smoking, alcohol consumption, and university degree (yes/no)

In separate analysis for history of and new onset depression (ESM Table S3), individuals with new onset depression had higher odds of poor versus good adherence (OR 1.74 [95% CI 1.05, 2.86], *P* = 0.03) after full adjustment, compared to those without new onset depression. In contrast, history of depression was not associated with the odds of poor (*P*= 0.3) or intermediate adherence ( *P*= 0.5) compared to good adherence. Similarly, when depression was defined exclusively based on diagnosis of depression (i.e., the ICD criteria recorded at a hospital inpatient or outpatient visits), no associations were observed between depression status and adherence categories (*P*= 0.5).

## Discussion

### Main findings

In the current study, we investigated the association between depression and refill adherence to cardioprotective medication in individuals with type 1 diabetes, and observed that depression was associated with higher odds of poor adherence, but not with intermediate adherence, when compared to good adherence. This implies that depression, particularly newly diagnosed depression, may contribute to poorer adherence to cardioprotective medications. We have previously reported that higher refill adherence to cardioprotective medication is associated with lower risk of CVD events [11]. Therefore, these individuals with depression, who are more likely to exhibit poor adherence, may be at increased risk of adverse cardiovascular outcomes. Our findings are consistent with earlier studies identifying depression as a risk factor for medication non-adherence in chronic diseases [14, 15], but our findings expand on this by distinguishing between adherence categories.

 We also found that new onset depression (i.e., having the first depressive episode during the 10-year follow-up period after the study baseline) was associated with poor adherence to cardioprotective medication, while an earlier history of depression was not. Of note, the adherence was assessed during the same 10-year follow-up period after the baseline visit. While our observational setup does not allow for causal conclusions, this might at least suggest that new onset depression poses a greater impact than earlier depressive episodes. Possibly these individuals lack coping strategies or adequate support, when the depression first appears [16]. In addition, in type 1 diabetes, the onset of depression can coincide with life transitions where diabetes management requires new adaptive efforts [17], potentially leading to poor adherence. Therefore, our findings emphasise the value of routinely assessing not only for a history of depression, but also the occurence of depressive symptoms during follow-up. Taken together, our observations highlight the importance of routine assessment not only for medication adherence, but also for the presence of depression at the regular consultations. Identifying and supporting individuals with depression, particularly those demonstrating poor adherence, is essential, as they may benefit the most from targeted interventions that integrate approaches addressing both mental health and adherence.

### Comparisons with previous studies

Previous studies have reported more symptoms of depression and higher use of antidepressants in adults with type 1 diabetes than in their age- and sex- matched counterparts without diabetes [18]. In addition, depression has been linked to worse glycaemic control [19], CVD outcomes [7], and diabetic complications [6]. We here report that over one-third of our cohort with cardioprotective medication had depression during a median study period of 15.9 years. Moreover, the same individuals, especially those with new onset depression after the study baseline, are more likely to have poor than good adherence to cardioprotective medications. In line with our findings, a cross-sectional study from the Netherlands found that less optimal medication intake and more perceived barriers correlate with depressive symptoms and diabetes-specific distress in individuals with type 1 diabetes [20]. In addition, depression has also been linked to a higher number of cardiovascular risk factors [21]. Even though, we observed lower adherence to cardioprotective medication in adults with depression compared to those without depression, it should be emphasised that the absolute differences in baseline metabolic risk factors were relatively small between those with and without depression. On the other hand, the metabolic risk factors were measured at a single time-point, which might not capture their changes over time. We were also unable to evaluate if the severity of depression influences these metabolic risk factors.

Type 1 diabetes is a complex disease, requiring not only intensive self-care to monitor and control blood glucose by insulin therapy, but also long-term therapies to control other risk factors [22]. Although depression has been connected with less favourable self-care behaviour in individuals with diabetes, it may also contribute to non-adherence to other medications and may therefore be a pathway to negative CVD outcomes [23]. Nevertheless, there is also evidence that diabetes distress per se is a clinically meaningful psychosocial stressor that could play a role in cardiovascular health in individuals with type 1 diabetes [24]. However, further studies are needed to investigate the role of diabetes distress in the development of CVD [24].

There is evidence that psychological or collaborative care interventions can improve medication adherence and clinical outcome in individuals with diabetes. Abbas et al. [25] recently demonstrated Cognitive Behaviour Therapy (CBT) to improve treatment adherence as well as psychological outcomes in individuals with type 2 diabetes. Also a meta-analysis found that collaborative care is associated with significantly better outcomes and adherence to antidepressants and oral hypoglycaemic agents in individuals with diabetes and comorbid depression [26]. Although poor adherence to treatment and worse health outcomes have been reported in depressive individuals with diabetes compared to those without depression [18], there is limited evidence whether treating depression alone may improve the adherence to treatment regimens and health outcomes in individuals with type 1 diabetes [19]. One small study, including 28 individuals with type 1 diabetes and depressive symptoms who received CBT showed improvement in depressive symptoms, but not in the HbA_1c_ levels [27]. Similarly, a meta-analysis of 21 randomised controlled trials in individuals with type 1 diabetes showed that psychological therapy can improve glycaemic control in children and adolescents, but not in adults [19]. Their findings suggest that interventions that target depression only may not be sufficient to improve adherence to self-care and diabetes treatment regimens. Therefore, a multidisciplinary approach that simultaneously focuses on depression and adherence to diabetes self-care regimens, including optimal medication intake, might be a feasible approach [28].

In such a multidisciplinary approach, the screening for symptoms of depression, distress, anxiety and other psychological issues has been recommended to be integrated into routine diabetes care at least by annually using standardized and validated tools (e.g., Patient Health Questionnaire [PHQ-9], Diabetes Distress Scales) [29, 30]. Importantly, diabetes care providers need to be trained to recognise psychosocial distress and to structurally collaborate with mental health professionals (e.g., psychologists, psychiatrists), ensuring access through coordinated referral pathways or integrated care [29, 30]. Going hand in hand, effective communication and clear information regarding the disease and its treatment are essential to improve adherence [31]. To improve an individual’s adherence to a long-term medication regimen, it is important to recognise factors that challenge the medication adherence, including depression. Other contributing barriers are social- and culture-specific barriers (e.g., lack of social network and support), health care and system-specific barriers (e.g., access to care, transitions of care, lack of communication with health professionals), as well as medication-specific barriers (e.g., symptoms, adverse events, polypharmacy, regimen complexity) [31].

### Strengths and limitations

The main strengths of this study include a careful characterisation of adults with type 1 diabetes, and the linkage of clinical data with comprehensive and high-quality national registry data, the long duration of follow-up, and the use of an objective measured refill adherence outcome, calculated from prescription records by the PDC method, which is widely accepted to measure long-term refill adherence [32]. A limitation with any prescription registry data is that such data do not provide information whether the medication was actually taken or not [33]. Another limitation is that we used purchases of antidepressants from a register, as a proxy of depression, which may have led to misclassification, as individuals with untreated or subclinical depression may not have been captured. Moreover, as the hospital register data lacked information on depression severity, and the DPR does not contain any information on actually prescribed dosages, our approach cannot assess the severity or duration of depressive symptoms. In addition, antidepressants may also be used to treat other conditions, such as anxiety, sleeping disorders, migraine, and neuropathic pain [34]. Consequently, some antidepressants may have been prescribed for indications unrelated to depression. As neuropathic pain is a typical complication in individuals with type 1 diabetes, we performed a sensitivity analysis by excluding medications that may potentially be used to treat neuropathic pain, but the significance of our finding did not change. Moreover, as the study only included individuals with at least two prescription fills, individuals with the poorest medication-taking behaviour, such as those, who filled a prescription once or not at all, would have been excluded. This may have led to a conservative estimation of the true association between depression and adherence. Furthermore, we did not collect data on socioeconomic status (beyond education) or diabetes distress, both of which are known to be linked with depression [30, 35]. Because these factors were not included in our analysis, some degree of residual confounding cannot be excluded. Finally, the observational nature of our study does not allow to assess any causality of the observed associations between depression and adherence to cardioprotective medications.

### Practical implications

In conclusion, over one-third of those adults with type 1 diabetes, who were prescribed cardioprotective medication, had also a diagnosis of depression or purchases of antidepressants during the study period. These individuals had higher odds of having poor rather than good adherence to cardioprotective therapy. Our findings highlight the importance of a multidisciplinary approach that simultaneously *focuses* on depression and the adherence to medications in clinical practice.

## Supplementary Information

Below is the link to the electronic supplementary material.

Supplementary Material 1

Supplementary Material 2

## Data Availability

Individual-level data for the study participants are not publicly available because of the restrictions due to the study consent provided by the participant at the time of data collection. The access to individual-level data, which is subject to local regulations, can be obtained upon reasonable request by contacting the FinnDiane Study Group board (manage@finndiane.fi). Upon approval, analysis needs to be performed on a user-specific local server (with protected access) and requires the applicant to sign non-disclosure and privacy agreements and comply with the General Data Protection Regulation.
