# Supplementary material for: Depression is associated with lower adherence to cardioprotective medications in adults with type 1 diabetes
Source: Acta Diabetol. 2025 Nov 18;63(2):259–66. doi: 10.1007/s00592-025-02610-3 (PMC12957039; doi:10.1007/s00592-025-02610-3)
Supplement: Supplementary file 1 — Supplementary Material 1 [file 592_2025_2610_MOESM1_ESM.pdf]

Electronic Supplementary Materials (ESM)

Depression is associated with lower adherence to cardioprotective medications in adults with type 1 diabetes

Acta Diabetologica

Raija Lithovius[1,2,3], Stefan Mutter[1,2,3], Erika B. Parente [1,2,3,4], Valma Harjutsalo[1,2,3,5], Per-Henrik Groop [1,2,3,6,7], Lena M. Thorn[1,2,3,8], Niina Sandholm[1,2,3], on behalf of the FinnDiane Study Group

[1]Folkhälsan Research Center, Helsinki, Finland; [2]Department of Nephrology, University of Helsinki and Helsinki University Hospital, Finland; [3]Research Program for Clinical and Molecular Metabolism, Faculty of Medicine, University of Helsinki, Finland; [4]Boehringer Ingelheim International GmbH, Ingelheim, Germany; [5]National Institute for Health and Welfare, Chronic Disease Prevention Unit, Helsinki, Finland; [6]Department of Diabetes, Central Clinical School, Monash University, Melbourne, VIC, Australia; [7]Baker Heart and Diabetes Institute, Melbourne, VIC, Australia; [8]Department of General Practice and Primary Health Care, University of Helsinki and Helsinki University Hospital, Helsinki, Finland

Corresponding author
Niina Sandholm, Folkhälsan Research Center, Haartmaninkatu 8 [C318b], 00290 Helsinki, FINLAND
Phone: +358 447881045
Email: niina.sandholm@helsinki.fi

Contents

ESM Table S1. Antihypertensive and lipid-lowering mediations. 2

ESM Table S2. Antidepressive medications potentially can be used for neuropathic pain 3

ESM Table S3. Multinominal regression analyses by depression status: a history of depression, new onset or diagnosed depression 4

ESM Table S4. Physicians and nurses at each of the FinnDiane centers participating in patient recruitment and characterization 5

ESM Table S1. Antihypertensive and lipid-lowering mediations. A national unique person identifier links the data in the FinnDiane with longitudinal prescription records from the Drug Prescription Register (DPR). Drugs were recorded according to the Anatomical Therapeutic Chemical (ATC) codes based on the Index Version 2024 (available from https://atcddd.fhi.no/atc_ddd_index/) and individuals taking combination pills were counted as taking separate classes for each drug. Drugs were classified into six subgroups:

Drug class	ATC code
RAAS (renin-angiotensin-aldosterone system) inhibitors	C09
Beta blockers	C07
Calcium channel blockers	C08, C09BB, C09DB, C07FB, C09BX01, C09DX01
Diuretics	C03, C09BA, C09DA, C07BB, C09BX01, C09DX01
Other antihypertensive drugs	C02AB01, C02AC, C02CA01
Lipid-lowering drugs	C10

ESM Table S2. Antidepressive medications (https://www.kaypahoito.fi/hoi50023). Medications with asterik (*) indicate medications which potentially can be used for neuropathic pain (https://www.kaypahoito.fi/hoi50103).

	ATC code	Medications used for depression	Treatment for neuropathic pain
Imipramine	N06AA02		
Clomipramine	N06AA04	X	
Trimipramine	N06AA06		
Amitriptyline *	N06AA09	X	X
Nortriptyline *	N06AA10	X	X
Doxepin	N06AA12		
Maprotiline	N06AA21		
Fluoxetine	N06AB03	X	
Citalopram	N06AB04	X	
Paroxetin	N06AB05	X	
Sertralin	N06AB06	X	
Fluvoxamin	N06AB08	X	
Essitalopram	N06AB10	X	
Mianserin	N06AX03	X	
Trazodone	N06AX05	X	
Moklobemid	N06AG02	X	
Mirtazapine	N06AX11	X	
Bupropion	N06AX12	X	
Venlafaxine *	N06AX16	X	X
Milnacipran	N06AX17		
Reboxetine	N06AX18		
Duloxetine *	N06AX21	X	X
Agomelatine	N06AX22	X	
Vortioxetine	N06AX26	X	
Amitriptyline and psycholeptics *	N06CA01	X	X

ESM Table S3. Multinominal regression analyses (i.e., adherence as outcome variable and depression status as explanatory variable) by depression status: A history of depression (at least one depressive episode between 1995 and the study baseline visit without a relapse after baseline), new onset (the first depressive episode during the 10-year follow-up period after the baseline) or any diagnosed depression (any diagnosis of depression according to the ICD criteria recorded at a hospital inpatient or outpatient visits)

Adherence class	History of depression no / yes, n	Model 1[a]		Model 2[b]		Model 3[c]	
		OR (95% CI), *P*	z	OR (95% CI), *P*	z	OR (95% CI), *P*	z
Good	535 / 59	Ref.		Ref.		Ref.	
Intermediate	794 / 69	0.79 (0.54, 1.13), 0.2	-1.2815	0.82 (0.56, 1.18), 0.3	-1.0735	0.87 (0.57, 1.33), 0.5	-0.6613
Poor	116 / 15	1.17 (0.64, 2.14), 0.6	0.5192	1.34 (0.72, 2.49), 0.3	0.9360	1.46 (0.71, 3.00), 0.3	1.0310
	New onset depression no / yes, n	Model 1[b]		Model 2[c]		Model 3[d]	
		OR (95% CI), *P*	z	OR (95% CI), *P*	z	OR (95% CI), *P*	z
Good	478 / 116	Ref.		Ref.		Ref.	
Intermediate	682/ 181	1.09 (0.84, 1.42), 0.5	0.6727	1.06 (0.82, 1.39), 0.7	0.4530	1.12 (0.84, 1.51), 0.4	0.7853
Poor	95 / 36	1.56 (1.01, 2.41), 0.04	2.0141	1.53 (0.98, 2.39), 0.06	1.8911	1.74 (1.05, 2.86), 0.03	2.1664
	Any diagnosed depression no / yes, n	Model 1[b]		Model 2[c]		Model 3[d]	
Good	519 / 75	Ref.		Ref.		Ref.	
Intermediate	764 / 99	0.90 (0.65, 1.23), 0.5	-0.6678	0.86 (0.62, 1.19), 0.4	-0.9261	0.89 (0.62, 1.28), 0.5	-0.6231
Poor	110 / 21	1.32 (0.78, 2.23), 0.3	1.0374	1.22 (0.71, 2.10), 0.5	0.7117	1.26 (0.68, 2.34), 0.5	0.7272

[a] Unadjusted
[b]Adjusted for age, sex, duration of diabetes, BMI, HbA_{1c}, kidney complications (moderate or severe albuminuria or eGFR <60 ml/min/1.73 m^2)
[c]Model 2 + smoking, alcohol consumption, and university degree (yes/no)

ESM Table S4. Physicians and nurses at each of the FinnDiane centers participating in patient recruitment and characterization

The Finnish Diabetic Nephropathy Study Center	Physicians and nurses
Anjalankoski Health Center	S.Koivula, T.Uggeldahl
Central Finland Central Hospital, Jyväskylä	T.Forslund, A.Halonen, A.Koistinen, P.Koskiaho, M.Laukkanen, J.Saltevo, M.Tiihonen
Central Hospital of Åland Islands, Mariehamn	M.Forsen, H.Granlund, A.-C.Jonsson, B.Nyroos
Central Hospital of Kanta-Häme, Hämeenlinna	P.Kinnunen, A.Orvola, T.Salonen, A.Vähänen
Central Hospital of Kymenlaakso, Kotka	R.Paldanius, M.Riihelä, L.Ryysy
Central Hospital of Länsi-Pohja, Kemi	H.Laukkanen, P.Nyländen, A.Sademies
Central Ostrobothnian Hospital District, Kokkola	S.Anderson, B.Asplund, U.Byskata, P.Liedes, M.Kuusela, T.Virkkala
City of Espoo Health Center:	
Espoonlahti	A.Nikkola, E.Ritola
Tapiola	M.Niska, H.Saarinen
Samaria	E.Oukko-Ruponen, T.Virtanen
Viherlaakso	A.Lyytinen
City of Helsinki Health Center:	
Puistola	H.Kari, T.Simonen
Suutarila	A.Kaprio, J.Kärkkäinen, B.Rantaeskola
Töölö	P.Kääriäinen, J.Haaga, A-L.Pietiläinen
City of Hyvinkää Health Center	S.Klemetti, T.Nyandoto, E.Rontu, S.Satuli-Autere
City of Vantaa Health Center:	
Korso	R.Toivonen, H.Virtanen
Länsimäki	R.Ahonen, M.Ivaska-Suomela, A.Jauhiainen
Martinlaakso	M.Laine, T.Pellonpää, R.Puranen
Myyrmäki	A.Airas, J.Laakso, K.Rautavaara
Rekola	M.Erola, E.Jatkola
Tikkurila	R.Lönnblad, A.Malm, J.Mäkelä, E.Rautamo
Heinola Health Center	P.Hentunen, J.Lagerstam
Helsinki University Central Hospital, Department of Medicine, Division of Nephrology	M.Feodoroff, D.Gordin, O.Heikkilä, K.Hietala, J.Fagerudd, M.Korolainen, L.Kyllönen, J.Kytö, S.Lindh, K.Pettersson-Fernholm, M.Rosengård-Bärlund, A.Sandelin, L.Thorn, J.Tuomikangas, T.Vesisenaho, J.Wadén
Herttoniemi Hospital, Helsinki	V.Sipilä
Hospital of Lounais-Häme, Forssa	T.Kalliomäki, J.Koskelainen, R.Nikkanen, N.Savolainen, H.Sulonen, E.Valtonen
Hyvinkää Hospital	L. Norvio, A.Hämäläinen
Iisalmi Hospital	E.Toivanen
Jokilaakso Hospital, Jämsä	A.Parta, I.Pirttiniemi
Jorvi Hospital, Helsinki University Central Hospital	S.Aranko, S.Ervasti, R.Kauppinen-Mäkelin, A.Kuusisto, T.Leppälä, K.Nikkilä, L.Pekkonen
Jyväskylä Health Center, Kyllö	K.Nuorva, M.Tiihonen
Kainuu Central Hospital, Kajaani	S.Jokelainen, K.Kananen, M.Karjalainen, P.Kemppainen, A-M.Mankinen, A.Reponen, M.Sankari
Kerava Health Center	H.Stuckey, P.Suominen
Kirkkonummi Health Center	A.Lappalainen, M.Liimatainen, J.Santaholma
Kivelä Hospital, Helsinki	A.Aimolahti, E.Huovinen
Koskela Hospital, Helsinki	V.Ilkka, M.Lehtimäki
Kotka Health Center	E.Pälikkö-Kontinen, A.Vanhanen
Kouvola Health Center	E.Koskinen, T.Siitonen
Kuopio University Hospital	E.Huttunen, R.Ikäheimo, P.Karhapää, P.Kekäläinen, M.Laakso, T.Lakka, E.Lapmpainen, L.Moilanen, S. Tanskanen, L.Niskanen, U.Tuovinen, I.Vauhkonen, E.Voutilainen, U.Tuovinen, I.Vauhkonen,
Kuusamo Health Center	T.Kääriäinen, E.Isopoussu
Kuusankoski Hospital	E.Kilkki, I.Koskinen, L.Riihelä
Laakso Hospital, Helsinki	T.Meriläinen, P.Poukka, R.Savolainen, N.Uhlenius
Lahti City Hospital	A.Mäkelä, M.Tanner
Lapland Central Hospital, Rovaniemi	L.Hyvärinen, K.Lampela, S.Pöykkö, T.Rompasaari, S.Severinkangas, T.Tulokas
Lappeenranta Health Center	P. Erola, L.Härkönen, P.Linkola, T.Pekkanen, I.Pulli, E.Repo
Lohja Hospital	T.Granlund, K.Hietanen, M.Porrassalmi, M.Saari, T.Salonen, M.Tiikkainen,
Länsi-Uusimaa Hospital, Tammisaari	I.-M.Jousmaa, J.Rinne
Loimaa Health Center	A.Mäkelä, P.Eloranta
Malmi Hospital, Helsinki	H.Lanki, S.Moilanen, M.Tilly-Kiesi
Mikkeli Central Hospital	A.Gynther, R.Manninen, P.Nironen, M.Salminen, T.Vänttinen
Mänttä Regional Hospital	I.Pirttiniemi, A-M.Hänninen

North Karelian Hospital, Joensuu	U-M.Henttula, P.Kekäläinen, M.Pietarinen, A.Rissanen, M.Voutilainen
Nurmijärvi Health Center	A.Burgos, K.Urtamo
Oulaskangas Hospital, Oulainen	E.Jokelainen, P-L.Jylkkä, E.Kaarlela, J.Vuolaspuro
Oulu Health Center	L.Hiltunen, R.Häkkinen, S.Keinänen-Kiukaanniemi
Oulu University Hospital	R.Ikäheimo
Päijät-Häme Central Hospital	H.Haapamäki, A.Helanterä, S.Hämäläinen, V.Ilvesmäki, H.Miettinen
Palokka Health Center	P.Sopanen, L.Welling
Pieksämäki Hospital	V.Sevtsenko, M.Tamminen
Pietarsaari Hospital	M-L.Holmbäck, B.Isomaa, L.Sarelin
Pori City Hospital	P.Ahonen, P.Merisalo, E.Muurinen, K.Sävelä
Porvoo Hospital	M.Kallio, B.Rask, S.Rämö
Raahe Hospital	A.Holma, M.Honkala, A.Tuomivaara, R.Vainionpää
Rauma Hospital	K.Laine, K.Saarinen, T.Salminen
Riihimäki Hospital	P.Aalto, E.Immonen, L.Juurinen
Salo Hospital	A.Alanko, J.Lapinleimu, P.Rautio, M.Virtanen
Satakunta Central Hospital, Pori	M.Asola, M.Juhola, P.Kunelius, M.-L.Lahdenmäki, P.Pääkkönen, M.Rautavirta
Savonlinna Central Hospital	T.Pulli, P.Sallinen, M.Taskinen, E.Tolvanen, T.Tuominen, H.Valtonen, A.Vartia, S-L. Viitanen
Seinäjoki Central Hospital	O.Antila, E.Korpi-Hyövälti, T.Latvala, E.Leijala, T.Leikkari, M.Punkari, , N.Rantamäki, H.Vähävuori
South Karelia Central Hospital, Lappeenranta	T.Ensala, E.Hussi, R.Härkönen, U.Nyholm, J.Toivanen
Tampere Health Center	A.Vaden, P.Alarotu, E.Kujansuu, H.Kirkkopelto-Jokinen, M.Helin, S.Gummerus, L.Calonius, T.Niskanen, T.Kaitala, T.Vatanen
Tampere University Hospital	P. Hannula, I.Ala-Houhala, R.Kannisto, T.Kuningas, P.Lampinen, M.Määttä, H.Oksala, T.Oksanen, A.Putila, H.Saha, K.Salonen, H.Tauriainen, S.Tulokas
Tiirismaa Health Center, Hollola	T.Kivelä, L.Petlin, L.Savolainen
Turku Health Center	A.Artukka, I.Hämäläinen, L.Lehtinen, E.Pyysalo, H.Virtamo, M.Viinikkala, M.Vähätalo
Turku University Central Hospital	K.Breitholz, R.Eskola, K.Metsärinne, U.Pietilä, P.Saarinen, R.Tuominen, S.Äyräpää
Vaajakoski Health Center	K.Mäkinen, P.Sopanen
Valkeakoski Regional Hospital	S.Ojanen, E.Valtonen, H.Ylönen, M.Rautiainen,T.Immonen
Vammala Regional Hospital	I.Isomäki, R.Kroneld, L.Mustaniemi, M.Tapiolinna-Mäkelä
Vaasa Central Hospital	S.Bergkulla, U.Hautamäki, V-A.Myllyniemi, I.Rusk
