# Supplementary material for: Depression is associated with lower adherence to cardioprotective medications in adults with type 1 diabetes
Source: Acta Diabetol. 2025 Nov 18;63(2):259–66. doi: 10.1007/s00592-025-02610-3 (PMC12957039; doi:10.1007/s00592-025-02610-3)
Supplement: Supplementary file 2 — Supplementary Material 2 [file 592_2025_2610_MOESM2_ESM.docx]

**Depression is associated with lower adherence to cardioprotective medications in adults with type 1 diabetes**

Raija Lithovius^1,2,3^, Stefan Mutter^1,2,3^, Erika B. Parente ^1,2,3,4^, Valma Harjutsalo^1,2,3,5^, Per-Henrik Groop ^1,2,3,6,7^, Lena M. Thorn^1,2,3,8^, Niina Sandholm^1,2,3^, on behalf of the FinnDiane Study Group

^1^Folkhälsan Research Center, Helsinki, Finland; ^2^Department of Nephrology, University of Helsinki and Helsinki University Hospital, Finland; ^3^Research Program for Clinical and Molecular Metabolism, Faculty of Medicine, University of Helsinki, Finland; ^4^Boehringer Ingelheim International GmbH, Ingelheim, Germany; ^5^National Institute for Health and Welfare, Chronic Disease Prevention Unit, Helsinki, Finland; ^6^Department of Diabetes, Central Clinical School, Monash University, Melbourne, VIC, Australia; *^7^*Baker Heart and Diabetes Institute, Melbourne, VIC, Australia ; ^8^Department of General Practice and Primary Health Care, University of Helsinki and Helsinki University Hospital, Helsinki, Finland

**Statements and Declarations**

**Declaration of interest**

SM received lecture honorarium from Encore Medial Education, PHG has received lecture honoraria from Astellas, Astra Zeneca, Bayer, Berlin Chemie, Boehringer Ingelheim, Eli Lilly, EloWater, Genzyme, Medscape, MSD, Mundipharma, Novartis, Novo Nordisk, PeerVoice, Sanofi and Sciarc.  PHG is an advisory board member for AbbVie, Astellas, Astra Zeneca, Bayer, Boehringer Ingelheim, Eli Lilly, Medscape, MSD, Mundipharma, Nestlé, Novartis, Novo Nordisk, and Sanofi. PHG has received investigator-initiated grants from Eli Lilly and Roche. EBP reports received lecture honorariums from Astra Zeneca, Sanofi during the last three years and has been an employee of Boehringer Ingelheim since February 2024. No other potential conflicts of interest relevant to this article were reported. The funding sources were not involved in the design or conduct of the study. All other authors declare that there is no duality of interest associated with this manuscript.

**Funding**

This study was supported by a grant from the Finnish Diabetes Research Foundation. The FinnDiane Study was supported by grants from Folkhälsan Research Foundation, Wilhelm and Else Stockmann Foundation, Liv och Hälsa Society, Medical Society of Finland, State funding for university-level health research by Helsinki University Hospital (TYH2023403), Sigrid Jusélius Foundation (220027, 250214), Finnish Foundation for Cardiovascular Research, and Novo Nordisk Foundation (NNF23OC0082732).
